# *Ophiocordycepsaphrophoridarum* sp. nov., a new entomopathogenic species from Guizhou, China

**DOI:** 10.3897/BDJ.9.e66115

**Published:** 2021-12-22

**Authors:** Yu Yang, Yuanpin Xiao, Gangjiang Yu, TingChi Wen, ChunYing Deng, Juan Meng, Zhenghua Lu

**Affiliations:** 1 School of liquor and food engineering, Guizhou University, Guiyang, China School of liquor and food engineering, Guizhou University Guiyang China; 2 The Engineering Research Center of Southwest Bio-Pharmaceutical Resources, Ministry of Education, Guizhou University, Guiyang, China The Engineering Research Center of Southwest Bio-Pharmaceutical Resources, Ministry of Education, Guizhou University Guiyang China; 3 Mushroom Research Center, School of agriculture, Guizhou University, Guiyang, China Mushroom Research Center, School of agriculture, Guizhou University Guiyang China; 4 Mae Fah Luang University, Chiang Rai, Thailand Mae Fah Luang University Chiang Rai Thailand; 5 State Key Laboratory Breeding Base of Green Pesticide and Agricultural Bioengineering, Key Laboratory of Green Pesticide and Agricultural Bioengineering, Ministry of Education, Guizhou University, Guiyang, China State Key Laboratory Breeding Base of Green Pesticide and Agricultural Bioengineering, Key Laboratory of Green Pesticide and Agricultural Bioengineering, Ministry of Education, Guizhou University Guiyang China; 6 Guizhou Institute of Biology, Guizhou Academy of Sciences, Guiyang, China Guizhou Institute of Biology, Guizhou Academy of Sciences Guiyang China; 7 Mushroom Research Center, School of agriculture, Guiyang, China Mushroom Research Center, School of agriculture Guiyang China

**Keywords:** one new taxon, morphology, *Ophiocordyceps*, multilocus phylogeny, taxonomy

## Abstract

**Background:**

*Ophiocordyceps* is the largest genus in the family Ophiocordicipitaceae, including many entomopathogenic species. In recent years, many species have been described in this genus, with a wide range of host insects. Entomopathogenic fungi include ecologically, economically and medicinally important species, but a large portion of their diversity remains to be discovered and described.

**New information:**

In this study, a new species, *Ophiocordycepsaphrophoridarum* sp. nov, parasitising *Aphrophoridae* sp. (Hemiptera) is proposed from China, based on evidence from morphology and molecular phylogenetic analyses. This species is characterised by fibrous, pigmented stromata, cylindrical asci and filiform ascospores. Compared to its closest relative, *O.tricentri*, the new species has wider perithecia and longer asci. Molecular phylogenetic analyses of a multilocus dataset (consisting of SSU, ITS, LSU, TEF1, RPB1 and RPB2) confirm its placement in *Ophiocordyceps*. *Ophiocordycepsaphrophoridarum* is morphologically described and illustrated with colour photographs. Morphological comparisons with closely-related species are also presented in tabulated format.

## Introduction

Insect-associated fungi represent a largely unknown and undescribed group; only 1.5% of these fungi have been reported (Mueller and Schmit 2007). In 2019, scientists determined 48 new species of animal-associated Sordariomycetes, including eight species of *Ophiocordyceps*, one of the best-known entomopathogenic genera (Cheek et al. 2020). The following year, 12 new species of *Ophiocordyceps* were described (Araujo et al. 2020, Tasanathai et al. 2020).

The genus *Ophiocordyceps* was proposed by Petch 1931) and was originally considered as a subgenus of *Cordyceps* (Kobayasi 1941, Kobayasi and Shimizu 1983). Sung et al. (2007b) established Ophiocordycipitaceae as a new family in Hypocreales with *Ophiocordyceps* as type genus. Due to the polyphyletic nature of *Cordyceps*, species formerly assigned to this genus had to be recombined in *Ophiocordyceps* (Sung et al. 2007a, Johnson et al. 2009). To date, *Ophiocordyceps* is the most speciose genus in Ophiocordycipitaceae with 289 accepted species (Index Fungorum, accessed 11 March 2021). Species of *Ophiocordyceps* are characterised by producing fibrous, hard, flexible, pigmented stromata and cylindrical asci with apical caps (Sung et al. 2007a, Ban et al. 2015, Maharachchikumbura et al. 2015, Wijayawardene et al. 2017, Xiao et al. 2019). The asexual morph of *Ophiocordyceps* is linked to *Hirsutella*, *Hymenostilbe*, *Paraisaria*, *Stibella* and *Syngliocladium* (Sung et al. 2007a, Thanakitpipattana et al. 2020) and known as *Hirsutella*-like and *Hymenostilbe*-like (Kepler et al. 2013, Maharachchikumbura et al. 2016, Maharachchikumbura et al. 2015).

Species in Ophiocordycipitaceae are found on a wide range of insect hosts; some taxa are host specific, such as *Ophiocordycepsunilateralis* sensu lato (De Bekker et al. 2014, Kobmoo et al. 2019). Blattaria, Coleoptera, Dermaptera, Diptera, Hemiptera, Hymenoptera, Isoptera, Lepidoptera, Megaloptera, Neuroptera, Odonata and Orthoptera are the insect orders most commonly reported to be associated with *Ophiocordyceps* (Evans et al. 2011, Luangsa-Ard et al. 2018, Araujo and Hughes 2019). The functional morphology of *Ophiocordyceps* is diverse and considered to be exclusively related to the host’s ecology and biology (Evans et al. 2011).

*Ophiocordyceps* has a pan-global distribution, but is most species-rich in the tropics and subtropics (Petch 1933, Petch 1937, Kobayasi 1941, Tzean et al. 1997, Ban et al. 2015). The Yuntai Mountain Nature Reserve, China, a dolomite karst landform, has become a hotspot for fungal diversity (Luo et al. 2013, Wen et al. 2015, Wen et al. 2017) and, in 2019, samples of *Ophiocordyceps* were collected that proved to be an undescribed species. Here, we formally describe this species, based on morphological study and the phylogenetic analysis of a multilocus dataset.

## Materials and methods

### Collection and morphological characteristics examination

Two fresh samples of *Ophiocordyceps*, parasitising *Aphrophoridae* sp. (Hemiptera), were collected in June 2019 from the broad-leaved forest in Yuntai Mountain Nature Reserve, Guizhou Province, China. The samples were dried with silica gel and then stored in plastic boxes in the Herbarium of Mae Fah Luang University (MFLU). For micro-morphological observations, ascomata were examined using a Motic SMZ 168 Series stereomicroscope (Motic, Xiamen, China). Structures were observed and measured after being sliced with a double-sided blade and placed into water. Microphotographs were taken using an Eclipe 80i compound microscope (Nikon, Tokyo, Japan), fitted with an EOS 600D camera (Canon, Tokyo, Japan). Measurements were made using the Tarosoft (R) Image Frame software v.0.9.7.

### DNA extraction, PCR amplification and determination of DNA sequences

DNA was extracted from dried fruiting bodies using the Fungal gDNA Kit (Biomiga, Sang Diego, CA, USA). We amplified the small and large subunits (SSU, LSU) of the ribosomal RNA gene, internal transcribed spacer region (ITS), translation elongation factor-1α (TEF1) and the largest and second-largest subunit of RNA polymerase II gene (RPB1, RPB2). The following primer pairs were used: NS1/NS4 for SSU, ITS4/ITS5 for ITS, LR0R/LR5 for LSU (Hopple and Vilgalys 1994, Vilgalys and Hester 1990, White et al. 1990), EF1-983F/EF1-2218R for TEF1 (Sung et al. 2007b), CRPB1A/RPB1Cr for RPB1 and fRPB2-6f/RPB2-7CR for RPB2 (Castlebury et al. 2004). The 25-μl PCR reaction volume contained 2 μl of DNA template, 8.5 μl of H_2_O, 1 μl of each forward reverse primer and 12.5 μl of 2× benchtoptm Taq Master Mix (Biomiga, San Diego, CA, USA). Cycling conditions were as follows: for SSU and LSU: initial denaturation at 94°C for 3 min; followed by 33 cycles at 94°C for 30 s, 51°C for 30 s and 72°C for 2 min; and final extension at 72°C for 10 min. For ITS: initial denaturation at 94°C for 3 min; followed by 33 cycles of 94°C for 30 s, 51°C for 50 s and 72°C for 45 s; and final extension at 72°C for 10 min. For TEF1: initial denaturation at 94°C for 3 min; followed by 33 cycles of 94°C for 30 s, 58°C for 50 s and 72°C for 1 min; and final extension at 72°C for 10 min. For RPB1: initial denaturation at 94°C for 3 min; followed by 33 cycles of 94°C for 1 min, 52°C for 1 min and 72°C for 1 min; and final extension at 72°C for 10 min. Lastly, for RPB2: initial denaturation at 94°C for 3 min; followed by 33 cycles of 94°C for 30 s, 54°C for 40 s and 72°C for 80 s; and final extension at 72°C for 10 min. Amplified PCR products were verified by 1% agarose gel electrophoresis, stained with ethidium bromide in 1× TBE. The PCR products were sequenced by Shanghai Shenggong Biological Engineering Co. (Hangzhou, Shanghai, China). Forward and reverse sequence reads were assembled and edited by BioEdit v.7.0.9 (Hall et al. 2011).

### Sequence alignment and phylogenetic analyses

Reference sequences (Suppl. materials 1, 2) were downloaded from NCBI GenBank, based on previous studies (Suppl. material 2, Ban et al. 2015, Crous et al. 2018, Kepler et al. 2012, Long et al. 2021, Sanjuan et al. 2015, Sung et al. 2007a, Xiao et al. 2019, Araujo et al. 2015, Araujo et al. 2018). Sequences were aligned with MAFFT v.7 (Katoh and Standley 2013, http://mafft.cbrc.jp/alignment/server/). TrimAl v.1.3 (Capella-Gutiérrez et al. 2009) was used for automated alignment trimming for poorly-aligned regions of each locus. *Tolypocladiuminflation* and *T.ophioglosoides* (Kepler et al. 2012, Schoch et al. 2012) were selected as outgroup taxa.

Maximum Likelihood (ML) analyses were performed using IQ-TREE 2 (Minh et al. 2020) under partitioned models; the built-in ModelFinder (Kalyaanamoorthy et al. 2017) was used to select appropriate models for each of the six loci. Branch support was estimated using 1000 ultrafast bootstrap (UFBoot2) replicates (Hoang et al. 2018). Bayesian Inference (BI) was determined by Markov Chain Monte Carlo (MCMC) sampling using MrBayes v.3.1.2 (Ronquist et al. 2012).The six loci were concatenated into a single dataset. BI was performed with six independent MCMC runs and trees were sampled every 100^th^ generation. The analyses were stopped after 5,000,000 generations when the average standard deviation of split frequencies was below 0.01. The convergence of the runs was checked using Tracer v.1.6 (Rambaut et al. 2018). The first 25% of the resulting trees were discarded as burn-in and posterior probabilities (PP) were calculated from the remaining sampled trees. The ML tree was visualised with FigTree v.1.4.0 (http://tree.bio.ed.ac.uk/software/figtree/).

## Taxon treatments

### Ophiocordyceps aphrophoridarum

Y.Yang, Y.P. Xiao & T.C. Wen sp. nov.

C721173A-BA99-503B-A924-63E050E0FD9F

IF558176

#### Materials

- **Type status:** Holotype. **Occurrence:** catalogNumber: MFLU 20–0641; recordedBy: Yu Yang; lifeStage: Telemorph; **Taxon:** scientificName: Ophiocordycepsaphrophoridarum; **Location:** country: China; stateProvince: Guizhou; locality: Qiandongnan, Shibing, Yuntaishan; verbatimElevation: 854m; locationRemarks: label transliteration: "Guizhou, Qiandongnan, Shibing, Yuntaishan, on Aphrophoridae sp., 19 June 2019, Yu Yang; verbatimCoordinates: 27°06′28.28″N,108°06′32.15″E; decimalLatitude: 27.107858; decimalLongitude: 108.108932; georeferenceProtocol: label; **Identification:** identifiedBy: Yuan-pin Xiao; dateIdentified: 2020
- **Type status:** Paratype. **Occurrence:** catalogNumber: MFLU 20–0642; recordedBy: Yu Yang; lifeStage: Telemorph; **Taxon:** scientificName: Ophiocordycepsaphrophoridarum; **Location:** country: China; stateProvince: Guizhou; locality: Qiandongnan, Shibing, Yuntaishan; verbatimElevation: 859m; locationRemarks: label transliteration: "Guizhou, Qiandongnan, Shibing, Yuntaishan, on Aphrophoridae sp., 19 June 2019, Yu Yang; verbatimCoordinates: 27°06′30.44″N,108°06′27.15″E; decimalLatitude: 27.108457; decimalLongitude: 108.107542; georeferenceProtocol: label; **Identification:** identifiedBy: Yuan-pin Xiao; dateIdentified: 2020

#### Description

Facesoffungi number: FoF09653

**Figure 1.**
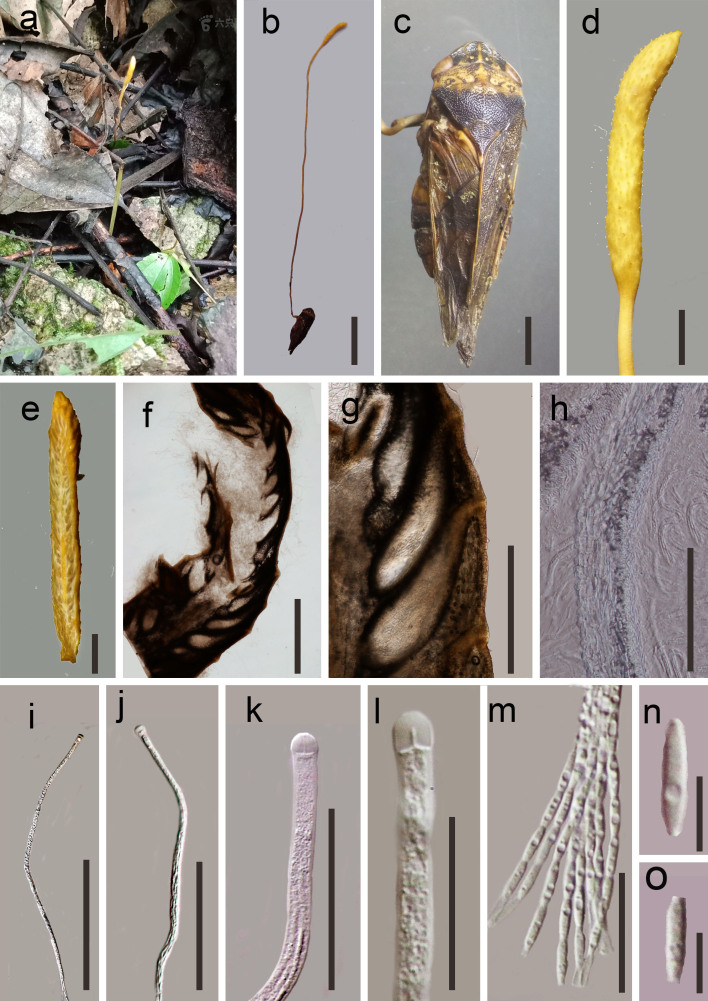
*Ophiocordycepsaphrophoridarum* (MFLU 20–0641, holotype): **a** Habitat **b** Overview of the host and stromata **c** Host **d** Stromata **e** Vertical section of the stroma **f**–**g** Section of ascomata **h** Peridium **i**–**j** Immature to mature asci **k**–**l** Apical cap of asci **m** Part of ascospores **n**–**o** Partspore. Scale bars: b = 10 mm, c–d = 5 mm, e–f = 1000 µm, g = 500 µm, i = 200 µm, j = 100 µm, k–m = 30 µm, n–o = 5 µm.

**Sexual morph: Stromata** 8–10 cm long, 0.5–3 mm diam., solitary, yellow, fibrous, unbranched, stipitate, slender. **Stipe** 7–8 cm long, 0.1–0.8 mm diam., cylindrical, with a fertile apex, yellow. **Fertile head** 1–2 cm long, 2–5mm diam., cylindrical to fusiform, differs from the stipe, yellow, single. **Perithecia** 638–798 × 108–178 μm (= 718 × 143 µm, n = 40), obliquely immersed, flask-shaped to elongated obpyriform. **Peridium** 26–68 µm (= 47 µm, n = 50) divided into two layers, hyaline, outer layer textura prismatica, inner layer textura porrecta. **Asci** 337–445 × 6.1–8.7 μm (= 391 × 7.4 µm, n = 60), 8-spored, hyaline, filiform, with a thick apex. **Apical cap** 5.1–8.2 × 3.6–5.2 μm (= 6.7 × 4.4 µm, n = 40), thick, with a small channel in the centre. **Ascospores** 258–315 × 3.1–5.5 μm (= 286.5 × 4.3 µm, n = 50), filiform, hyaline, multiseptate, easily breaking into secondary ascospores. **Partspore** 6.4–8.8 × 1.4–2.4 μm (= 7.6 × 1.9 µm, n = 90), fusoid, 1-celled, hyaline, smooth–walled. **Asexual morph**: Undetermined (Fig. 1)

#### Etymology

Referring to the host, Aphrophoridae sp.

#### Distribution

Thus far only known from China.

#### Host

Aphrophoridae sp. (Hemiptera), collected from the underside of leaves litter, stromata growing from the prothorax.

## Analysis

### Phylogenetic analyses

**Figure 2.**
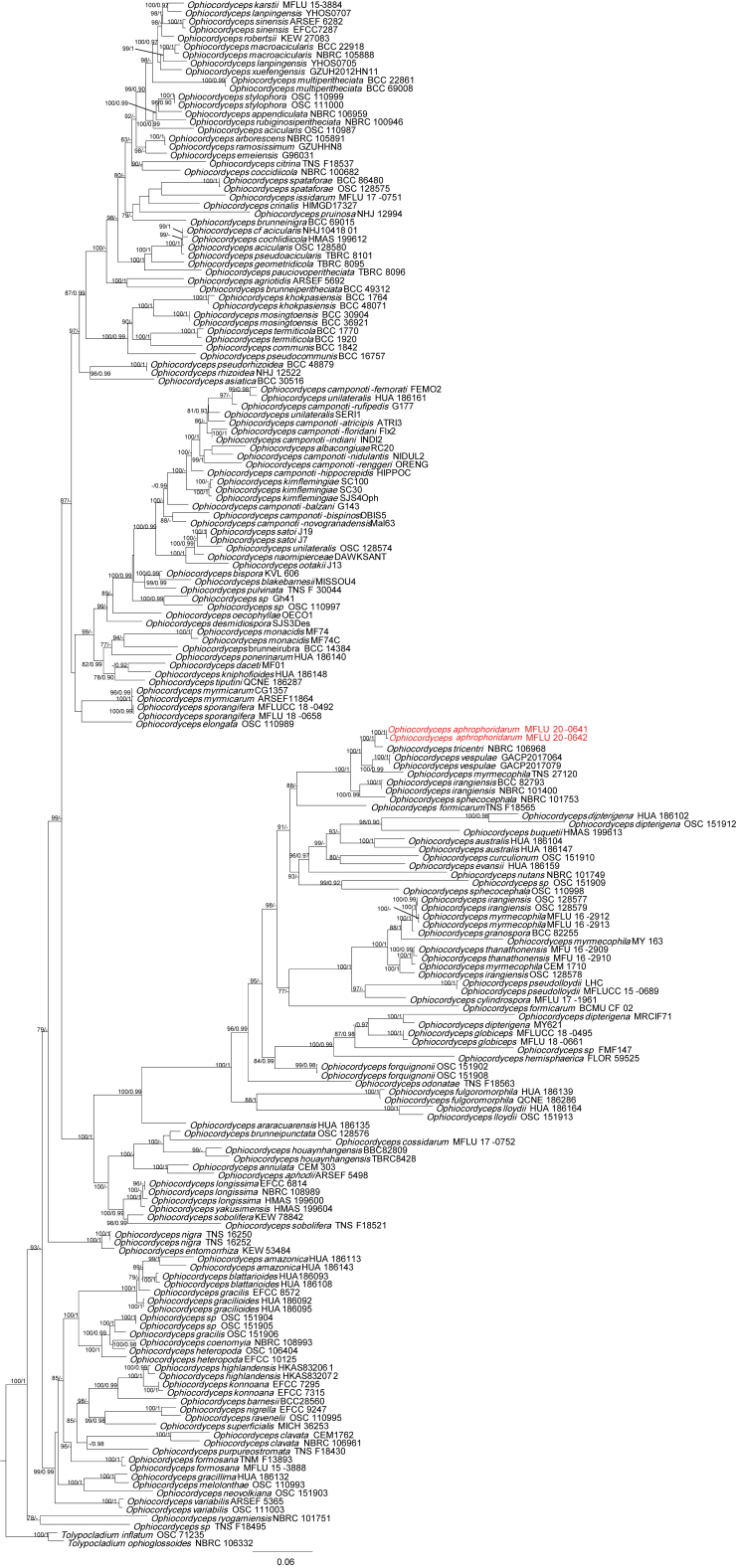
Phylogeny of *Ophiocordyceps* reconstructed from a six-locus dataset (ITS, SSU, LSU, TEF1, RPB2 and RPB1). The topology is the result of ML inference performed in IQ-TREE. The tree is rooted with both *Tolypocladiuminflatum* and *T.ophioglossoides*. MLBS ≥ 75 and BIPP ≥ 0.90 are presented above branches. The new species is highlighted in red.

A total of 185 sequences, representing 128 species of Ophiocordycipitaceae, were downloaded from GenBank. The final alignment length was 4412 characters, representing 185 taxa (822 for LSU, 481 for ITS, 919 for SSU, 918 for TEF1, 536 for RPB1 and 736 for RPB2) (Suppl. materials 1, 2). Tree topology of the IQ-TREE analysis was similar to the one from the Bayesian analyses. The best-scoring ML (-lnL = 81595.8951) is shown in Fig. 2.

## Discussion

The Yuntai Mountain Nature Reserve, situated in Shibing County, Guizhou Province, China, is a dolomite karst landform. The Reserve is home to 106 species of macrofungi (Luo et al. 2013), including two species of *Metacordyceps* that are currently only known from the holotype locality (Wen et al. 2015, Wen et al. 2017) Here, we present a new entomopathogenic species, *O.aphrophoridarum*, from the same Reserve.

*Ophiocordycepsaphrophoridarum* was phylogenetically retrieved as a sister species of *O.tricentri*, in a maximum supported clade with *O.irangiensis*, *O.myrmecophila*, *O.sphecocephala* and *O.vespulae* (Fig. 2). The sequences of six loci of *O.tricentri*, *O.irangiensis*, *O.sphecocephala* and *O.vespulae* share between 86–94% identity with *O.aphrophoridarum* in their ITS, 94–99% in SSU), 99–100% in LSU, 97–99% in TEF1, 94–95% in RPB1 and 95–97% in RPB2.

Both *O.irangiensis* and *O.myrmecophila* have Formicinae spp. (Hymenoptera) as host (Hywel-Jones 1996), whereas the host of *O.aphrophoridarum* is Aphrophoridae sp. (Hemiptera). Morphologically, *O.aphrophoridarum* differs from *O.irangiensis* in its smaller ascomata, shorter asci and shorter partspores (Hywel-Jones 1996). *Ophiocordycepsaphrophoridarum* differs from *O.myrmecophila* in terms of having smaller ascomata, shorter asci and longer partspores (Hywel-Jones 1996). The host of *O.sphecocephala* is *Vespula* sp. (Hymenoptera) (Hywel-Jones 1995). This species produces larger ascomata, longer asci and longer partspores compared to *O.aphrophoridarum* (Shrestha and Sung 2005). Additionally, *O.vespulae* has *Vespula* sp. as host (Hymenoptera) and is distinct from the new species by its longer asci and partspores (Long et al. 2021).

*Ophiocordycepstricentri* is phylogenetically most closely related to the new species and it has similar morphological characters. *Ophiocordycepstricentri* was initially described as *Cordycepstricentri* from Japan. It is characterised by stipitate stroma with a yellow fusoid fertile head (Yasuda 1922, Table 1). The host of *C.tricentri* was initially identified as *Tricentrus* sp. (Hemiptera, Membracidae), but later corrected to *Aphrophoraintermedia* (Hemiptera, Aphrophoridae) (Yasuda 1922). Later, *Aphrophoraflavomaculata*, *Aphrophorarugosa* and *Peuceptyelusmedius* were reported as the hosts of *C.tricentri* (Kobayasi 1941, Shrestha 2017). Additionally, another species, *Cordycepsaphrophorae*, was synonymised with *C.tricentri* (Yasuda 1922, Lim and Kim 1973). Shrestha and Sung 2005) recorded *Cordycepstricentri* obtained from Nepal, but presented no molecular data (Table 1). Following molecular phylogenetic analyses, *C.tricentri* was transferred to *Ophiocordyceps* (Sung et al. 2007b). Ban et al. 2015) presented sequence data of *O.tricentri* from strain NBRC 106968, but did not provide morphological information. It is clear that more data are needed to fully understand the species limits with regards to *O.tricentri*. The new species, *O.aphrophoridarum*, is morphologically similar to *O.tricentri*, but can be recognised by its longer and finer stromata and much longer asci (Yasuda 1922, Shrestha and Sung 2005, Table 1).

In conclusion, there is sufficient evidence from both morphology and molecular phylogenetic analyses to support *O.aphrophoridarum* as a new species of *Ophiocordyceps*.

## Supplementary Material

XML Treatment for Ophiocordyceps aphrophoridarum

E2D27493-FD8D-54D0-BB5A-48F336B820DC 10.3897/BDJ.9.e66115.suppl1 Supplementary material 1 Sources of isolates and GenBank accession numbers Data type MS Word Brief description Sources of isolates and GenBank accession numbers used in this study File: oo_560199.docx https://binary.pensoft.net/file/560199 Yu Yang, Yuan-pin Xiao, Gang-jiang Yu, Juan Meng, Zheng-hua Lu, Chun-ying Deng, Ting-chi Wen

C4AA4898-75A0-59F0-A1E2-F11B8F158881 10.3897/BDJ.9.e66115.suppl2 Supplementary material 2 References for GenBank accession numbers Data type MS Word Brief description References for GenBank accession numbers used in this study File: oo_559657.docx https://binary.pensoft.net/file/559657 Yu Yang

## Figures and Tables

**Table 1.** Synopsis of closely-related *Ophiocordyceps* species. Measurements in µm.

**Species**	*O.aphrophoridarum*	*O.tricentri* (holotype)	*O.tricentri* (EFCC 7251, 7252)
**Distribution**	China	Japan	Nepal
**Stromata (mm)**	Clavated, branched or unbranched, 80–100 × 0.5–1.2	Fusoid, yellow, unbranched	Solitary, yellow, 50–60 × 1–1.5
**Fertile heads (mm)**	Yellow single, allantoideus, 10–20 × 2–5		Ovoid, 50–60 × 1–1.5
**Perithecia (μm)**	Obliquely buried, ovoid to elongated pyriform, 638–798 × 108–178		Immersed, ovoid, 550–650 × 110–120
**Asci (μm)**	8-spored, hyaline, filiform, 337–445 × 6.1–8.7	Cylindrical, 120 × 5–6	300–320 × 5
**Partspores (μm)**	Fusoid, 1-celled, straight, hyaline, 6.4–8.8 × 1.4–2.4	Fusoid, smooth, 1-celled, hyaline, 8–10 × 1.5	
**References**	This study	Yasuda 1922	Shrestha and Sung 2005
