# Supplementary material for: Ophiocordycepsaphrophoridarum sp. nov., a new entomopathogenic species from Guizhou, China
Source: Biodivers Data J. 2021 Dec 22;9:e66115. doi: 10.3897/BDJ.9.e66115 (PMC8716513; doi:10.3897/BDJ.9.e66115)
Supplement: Supplementary material 1 — Sources of isolates and GenBank accession numbers [file bdj-09-e66115-s001.docx]

| Suppl Sources of isolates and GenBank accession numbers used in this study | | | | | | | | |
| --- | --- | --- | --- | --- | --- | --- | --- | --- |
| **Taxa names** | **Specimen/** **Strain** **number** | **GenBank accession numbers** | | | | | | **References** |
|  |  | **ITS** | **LSU** | **TEF1-α** | **RPB2** | **SSU** | **RPB1** |  |
| *Ophiocordyceps acicularis* | OSC 110987 |  | EF468805 | EF468744 |  | EF468950 | EF468852 | Sung et al. 2007a |
| *O. acicularis* | OSC 128580 | JN049820 | DQ518757 | DQ522326 | DQ522423 | DQ522543 | DQ522371 | Kepler et al. 2012 |
| *O. agriotidis* | ARSEF 5692 | JN049819 | DQ518754 | DQ522322 | DQ522418 | DQ522540 | DQ522368 | Ban et al. 2015 |
| *O. albacongiuae* | RC20 |  |  | KX713670 |  | KX713633 |  | Araújo et al. 2018 |
| *O.amazonica* | HUA 186113 |  | KJ917572 |  | KM411980 | KJ917566 | KP212903 | Sanjuan et al. 2015 |
| *O. amazonica* | HUA 186143 |  | KJ917571 | KM411989 | KM411982 | KJ917562 | KP212902 | Sanjuan et al. 2015 |
| *O. annulata* | CEM 303 |  |  | KJ878962 |  | KJ878915 | KJ878995 | Quandt et al. 2014 |
| *O. aphodii* | ARSEF 5498 |  | DQ518755 | DQ522323 | DQ522419 | DQ522541 |  | Spatafora et al. 2007 |
| *O. appendiculata* | NBRC 106959 | JN943325 | JN941412 | AB968578 | AB968540 | JN941729 | JN992463 | Ban et al. 2015 |
| *O. araracuarensis* | HUA 186135 |  | KC610769 | KC610738 | KC610716 | KC610788 | KF658665 | Sanjuan et al. 2015 |
| *O. arborescens* | NBRC 105891 | AB968398 | AB968414 | AB968572 | AB968534 | AB968386 |  | Ban et al. 2015 |
| *O. asiatica* | BCC 30516 | MH754722 | MH753675 | MK284263 | MK214091 |  | MK214105 | Tasanathai et al. 2019 |
| *O. australis* | HUA 186147 | KF937351 | KC610764 | KC610734 |  | KC610784 | KF658678 | Sanjuan et al. 2015 |
| *O. australis* | HUA 186104 |  | KC610763 | KC610733 | KC610713 | KC610783 |  | Sanjuan et al. 2015 |
| *O. barnesii* | BCC28560 |  |  |  | EU418599 | EU408776 | EU408773 | Luangsa-ard et al. 2010 |
| *O. bispora* | KVL 606 |  | AF009654 |  |  | KX713641 | KX713716 | Suhet et al. 1998 |
| *O. blakebarnesii* | MISSOU4 |  | KX713609 | KX713685 |  | KX713642 | KX713715 | Araújo et al. 2018 |
| *O. blattarioides* | HUA186093 |  | KJ917570 | KM411992 |  | KJ917559 | KP212910 | Sanjuan et al. 2015 |
| *O. blattarioides* | HUA 186108 |  | KJ917569 |  | KM411984 | KJ917558 | KP212912 | Sanjuan et al. 2015 |
| *O. brunneinigra* | BCC 69015 |  | MF614653 | MF614637 | MF614680 |  |  | Luangsa-Ard et al. 2018 |
| *O. brunneiperitheciata* | BCC 49312 |  | MF614660 | MF614642 | MF614686 |  |  | Luangsa-Ard et al. 2018 |
| *O. brunneipunctata* | OSC 128576 |  | DQ518756 | DQ522324 | DQ522420 | DQ522542 | DQ522369 | Spatafora et al. 2007 |
| *O. brunneirubra* | BCC 14384 | MH754736 | MH753690 | GU797121 | MK751468 |  | MK751465 | Tasanathai et al. 2019 |
| *O. buquetii* | HMAS 199613 |  | KJ878904 | KJ878984 |  | KJ878939 | KJ879019 | Quandt et al. 2014 |
| *O. camponoti–atricipis* | ATRI3 |  |  | KX713677 |  | KX713666 |  | Araújo et al. 2018 |
| *O. camponoti–balzani* | G143 |  | KX713595 | KX713690 |  | KX713658 | KX713705 | Araújo et al. 2018 |
| *O. camponoti–bispinosi* | OBIS5 |  | KX713616 | KX713693 |  | KX713636 | KX713721 | Araújo et al. 2018 |
| *O. camponoti–femorati* | FEMO2 |  | KX713590 | KX713678 |  | KX713663 | KX713702 | Araújo et al. 2018 |
| *O. camponoti–floridani* | Flx2 |  | KX713592 | KX713674 |  |  |  | Araújo et al. 2018 |
| *O. camponoti–hippocrepidis* | HIPPOC |  | KX713597 | KX713673 |  | KX713655 | KX713707 | Araújo et al. 2018 |
| *O. camponoti–indiani* | INDI2 |  | KX713598 |  |  | KX713654 |  | Araújo et al. 2018 |
| *O. camponoti–nidulantis* | NIDUL2 |  | KX713611 | KX713669 |  | KX713640 | KX713717 | Araújo et al. 2018 |
| *O. camponoti–novogranadensis* | Mal63 |  | KX713603 |  |  | KX713648 |  | Araújo et al. 2018 |
| *O. camponoti–renggeri* | ORENG |  | KX713617 | KX713671 |  | KX713634 |  | Araújo et al. 2018 |
| *O. camponoti–rufipedis* | G177 |  | KX713596 | KX713680 |  | KX713657 |  | Araújo et al. 2018 |
| *O. cf acicularis* | NHJ10418 01 | GU723765 |  | GU797116 |  |  |  | Luangsa-Ard et al.2011 |
| *O. citrina* | TNS F18537 |  | KJ878903 | KJ878983 |  |  |  | Quandt et al. 2014 |
| *O. clavata* | NBRC 106961 | JN943327 | JN941414 | AB968586 | AB968547 | JN941727 | JN992461 | Schoch et al. 2012 |
| *O. clavata* | CEM1762 |  | KJ878882 | KJ878963 |  | KJ878916 | KJ878996 | Quandt et al. 2014 |
| *O. coccidiicola* | NBRC 100682 | AB968404 | AB968419 | AB968583 | AB968545 | AB968391 |  | Ban et al. 2015 |
| *O. cochlidiicola* | HMAS 199612 |  | KJ878884 | KJ878965 |  |  |  | Quandt et al. 2014 |
| *O. coenomyia* | NBRC 108993 | AB968396 | AB968412 | AB968570 | AB968532 | AB968384 |  | Ban et al. 2015 |
| *O. communis* | BCC 1842 | MH754726 | MH753680 | MK284266 | MK214096 |  | MK214110 | Tasanathai et al. 2019 |
| *O. cossidarum* | MFLU 17**–**0752 |  | MF398187 |  |  | MF398186 | MF928404 | Hyde et al. 2017 |
| *O. crinalis* | HIMGD17327 | EU149926 |  |  |  |  |  | Zhang et al. 2007 |
| *O. curculionum* | OSC 151910 |  | KJ878885 |  |  | KJ878918 | KJ878999 | Quandt et al. 2014 |
| *O. cylindrospora* | MFLU 17**–**1961 | MG553635 | MG553652 |  | MG647029 |  |  | Hyde et al. 2018 |
| *O. daceti* | MF01 |  | KX713604 | KX713667 |  |  |  | Araújo et al. 2018 |
| *O. desmidiospora* | SJS3Des |  | MH536514 | MN785129 |  | MH536515 | MN785131 | Saltamachia et al.2020 |
| *O. dipterigena* | MRCIF71 | EU573346 |  |  |  |  |  | Freire 2015 |
| *O. dipterigena* | OSC 151912 |  | KJ878887 | KJ878967 |  | KJ878920 | KJ879001 | Quandt et al. 2014 |
| *O. dipterigena* | HUA 186102 |  | KJ917568 |  | KC610715 | KC610787 | KF658664 | Quandt et al. 2014 |
| *O. dipterigena* | MY621 | GU723764 |  | GU797126 |  |  |  | Luangsa-ard et al. 2011 |
| *O. elongata* | OSC 110989 |  | EF468808 | EF468748 |  |  | EF468856 | Sung et al. 2007a |
| *O. emeiensis* | G96031 | AJ309347 |  |  |  |  |  | Liu et al. 2002 |
| *O. entomorrhiza* | KEW 53484 | JN049850 | EF468809 | EF468749 | EF468911 | EF468954 | EF468857 | Quandt et al. 2014 |
| *O. evansii* | HUA 186159 | KP200889 | KC610770 | KC610736 |  | KC610796 | KP212916 | Sanjuan et al. 2015 |
| *O. formicarum* | TNS F18565 |  | KJ878888 | KJ878968 | KJ878946 | KJ878921 | KJ879002 | Quandt et al. 2014 |
| *O. formicarum* | BCMU CF 02 | AB222679 |  |  |  |  |  | Freire 2015 |
| *O. formosana* | TNM F13893 |  |  | KJ878956 | KJ878943 | KJ878908 | KJ878988 | Quandt et al. 2014 |
| *O. formosana* | MFLU 15**–**3888 |  |  | KU854949 |  |  |  | Li et al. 2016 |
| *O. forquignonii* | OSC 151902 |  | KJ878876 |  | KJ878945 | KJ878912 | KJ878991 | Quandt et al. 2014 |
| *O. forquignonii* | OSC 151908 |  | KJ878889 |  | KJ878947 | KJ878922 | KJ879003 | Quandt et al. 2014 |
| *O. fulgoromorphila* | QCNE 186286 |  | KC610759 |  |  | KC610793 |  | Luangsa-ard et al. 2011 |
| *O. fulgoromorphila* | HUA 186139 |  | KC610760 | KC610729 | KC610719 | KC610794 | KF658676 | Sanjuan et al. 2015 |
| *O. geometridicola* | TBRC 8095 |  | MF614648 | MF614632 | MF614679 |  | MF614663 | Luangsa-Ard et al. 2018 |
| *O. globiceps* | MFLUCC 18**–**0495 | MH725815 | MH725829 | MH727387 |  | MH725811 |  | Xiao et al. 2019 |
| *O. globiceps* | MFLU 18**–**0661 | MH725816 | MH725830 | MH727388 |  | MH725812 |  | Xiao et al. 2019 |
| *O. gracilioides* | HUA 186095 |  |  | KM411994 |  | KJ917556 | KP212914 | Araújo et al. 2018 |
| *O. gracilioides* | HUA 186092 |  | KJ130992 |  |  | KJ917555 | KP212915 | Araújo et al. 2018 |
| *O. gracilis* | OSC 151906 |  | KJ878890 | KJ878969 |  | KJ878923 |  | Quandt et al. 2014 |
| *O. gracilis* | EFCC 8572 | JN049851 | EF468811 | EF468751 | EF468912 | EF468956 | EF468859 | Kepler et al. 2012 |
| *O. gracillima* | HUA 186132 | KF937353 | KC610768 | KC610744 |  |  | KF658666 | Sanjuan et al. 2015 |
| *O. granospora* | BCC 82255 | MH028143 | MH028156 | MH028183 | MH028177 |  | MH028168 | Araújo et al. 2018 |
| *O. hemisphaerica* | FLOR 59525 | KX197233 |  |  |  |  |  | Hyde et al. 2016 |
| *O. heteropoda* | EFCC 10125 | JN049852 | EF468812 | EF468752 | EF468914 | EF468957 | EF468860 | Kepler et al. 2012 |
| *O. heteropoda* | OSC 106404 |  | AY489722 | AY489617 |  | AY489690 | AY489651 | Castlebury et al. 2004 |
| *O. highlandensis* | HKAS83207 2 |  |  |  | KM581281 |  |  | Yang et al. 2015 |
| *O. highlandensis* | HKAS83206 1 |  |  |  | KM581278 |  |  | Yang et al. 2015 |
| *O. houaynhangensis* | BBC82809 | MH092892 | MH092908 | MH092899 |  |  |  | Crous et al. 2018 |
| *O. houaynhangensis* | TBRC8428 | MH092891 | MH092902 | MH092894 |  |  |  | Crous et al. 2018 |
| *O. irangiensis* | BCC 82793 | MH028141 |  | MH028185 | MH028173 |  | MH028163 | Araújo et al. 2018 |
| *O. irangiensis* | OSC 128578 | JN049833 | DQ518770 | DQ522345 | DQ522445 | DQ522556 | DQ522391 | Spatafora et al. 2007 |
| *O. irangiensis* | OSC 128577 | JN049823 | DQ518760 | DQ522329 | DQ522427 | DQ522546 | DQ522374 | Spatafora et al. 2007 |
| *O. irangiensis* | OSC 128579 |  | EF469076 | EF469060 | EF469107 | EF469123 | EF469089 | Sung et al. 2007a |
| *O. irangiensis* | NBRC 101400 | JN943335 | JN941426 |  |  | JN941715 | JN992449 | Schoch et al. 2012 |
| *O. issidarum* | MFLU 17**–**0751 | MF398185 | MF398188 |  |  |  |  | Hyde et al. 2017 |
| *O. karstii* | MFLU 15**–**3884 |  |  | KU854945 |  | KU854952 | KU854943 | Li et al. 2016 |
| *O. khokpasiensis* | BCC 48071 | MH754728 | MH753682 | MK284269 |  |  | MK214112 | Tasanathai et al. 2019 |
| *O. khokpasiensis* | BCC 1764 | MH754730 | MH753684 | MK284271 | MK214098 |  | MK214114 | Tasanathai et al. 2019 |
| *O. kimflemingiae* | SC30 |  | KX713622 | KX713699 |  | KX713629 | KX713727 | Araújo et al. 2018 |
| *O. kimflemingiae* | SC100 |  | KX713624 | KX713696 |  |  | KX713725 | Araújo et al. 2018 |
| *O. kimflemingiae* | SJS4Oph |  | MH536516 | MN785130 |  |  | MN785132 | Saltamachia et al. 2020 |
| *O. kniphofioides* | HUA 186148 |  |  | KC610739 | KC610717 | KC610790 | KF658667 | Sanjuan et al. 2015 |
| *O. konnoana* | EFCC 7295 |  |  |  | EF468915 | EF468958 | EF468862 | Sanjuan et al. 2015 |
| *O. konnoana* | EFCC 7315 |  |  | EF468753 | EF468916 | EF468959 | EF468861 | Sung et al. 2007a |
| *O. lanpingensis* | YHOS0707 |  | KC417461 | KC417463 |  | KC417459 | KC417465 | Chen et al. 2013 |
| *O. lanpingensis* | YHOS0705 |  | KC417460 | KC417462 | KC456333 | KC417458 | KC417464 | Chen et al. 2013 |
| *O. lloydii* | HUA 186164 | KP200892 |  | KC610741 |  | KC610805 |  | Sanjuan et al. 2015 |
| *O. lloydii* | OSC 151913 |  | KJ878891 | KJ878970 | KJ878948 | KJ878924 | KJ879004 | Quandt et al. 2014 |
| *O. longissima* | EFCC 6814 |  | EF468817 | EF468757 |  |  | EF468865 | Kepler et al. 2012 |
| *O. longissima* | NBRC 108989 | AB968407 | AB968421 | AB968585 |  | AB968394 |  | Sanjuan et al. 2015 |
| *O. longissima* | HMAS 199600 |  |  | KJ878972 | KJ878949 |  |  | Quandt et al. 2014 |
| *O. macroacicularis* | BCC 22918 |  | MF614655 | MF614639 | MF614675 |  | MF614669 | Araújo et al. 2018 |
| *O. macroacicularis* | NBRC 105888 | AB968401 | AB968417 | AB968575 | AB968537 | AB968389 |  | Ban et al. 2015 |
| *O. melolonthae* | OSC 110993 |  | DQ518762 | DQ522331 |  | DQ522548 | DQ522376 | Spatafora et al. 2007 |
| *O. monacidis* | MF74C |  | KX713606 |  |  | KX713646 |  | Araújo et al. 2018 |
| *O. monacidis* | MF74 |  | KX713605 |  |  | KX713647 | KX713712 | Araújo et al. 2018 |
| *O. mosingtoensis* | BCC 30904 | MH754732 | MH753686 | MK284273 | MK214100 |  | MK214115 | Tasanathai et al. 2019 |
| *O. mosingtoensis* | BCC 36921 | MH754731 | MH753685 | MK284272 | MK214099 |  | MK214116 | Tasanathai et al. 2019 |
| *O. multiperitheciata* | BCC 22861 |  | MF614656 | MF614640 | MF614683 |  | MF614670 | Araújo et al. 2018 |
| *O. multiperitheciata* | BCC 69008 |  | MF614657 | MF614641 | MF614682 |  |  | Luangsa-Ard et al. 2018 |
| *O. myrmecophila* | MY 163 | GU723759 |  | GU797132 |  |  |  | Luangsa-Ard et al. 2011 |
| *O. myrmecophila* | CEM 1710 |  |  | KJ878974 |  | KJ878928 | KJ879008 | Quandt et al. 2014 |
| *O. myrmecophila* | TNS 27120 |  | KJ878895 | KJ878975 |  | KJ878929 | KJ879009 | Quandt et al. 2014 |
| *O. myrmecophila* | MFLU 16**–**2912 | MF351726 | MF372585 | MF372759 |  | MF351730 |  | Xiao et al. 2017 |
| *O. myrmecophila* | MFLU 16**–**2913 | MF351727 | MF372586 |  |  |  |  | Xiao et al. 2017 |
| *O. myrmicarum* | ARSEF11864 | JX566954 | JX566965 | JX566973 |  | KJ680150 | KJ680151 | Simmons et al. 2015 |
| *O. myrmicarum* | CG1357 |  | MG922561 | MG922554 |  | MG922559 | MG922556 | Torres et al. 2018 |
| *O. neovolkiana* | OSC 151903 |  | KJ878896 | KJ878976 |  | KJ878930 | KJ879010 | Quandt et al. 2014 |
| *O. nigra* | TNS 16252 |  | KJ878906 | KJ878986 |  | KJ878941 |  | Quandt et al. 2014 |
| *O. nigra* | TNS 16250 |  |  | KJ878987 |  | KJ878942 | KJ879021 | Quandt et al. 2014 |
| *O. nigrella* | EFCC 9247 | JN049853 | EF468818 | EF468758 | EF468920 | EF468963 | EF468866 | Sung et al. 2007a |
| *O. nutans* | NBRC 101749 | AB968408 | JN941429 | AB968589 | AB968550 | JN941712 | JN992446 | Ban et. al. 2015 |
| *O. odonatae* | TNS F18563 | AB104725 | KJ878877 |  |  |  | KJ878992 | Ito and Hirano 1997 |
| *O. oecophyllae* | OECO1 |  |  |  |  | KX713635 |  | Araújo et al. 2018 |
| *O. ootakii* | J13 |  | KX713600 | KX713681 |  | KX713652 | KX713708 | Araújo et al. 2018 |
| *O. pauciovoperitheciata* | TBRC 8096 |  | MF614649 | MF614636 | MF614672 |  | MF614665 | Luangsa-Ard et al. 2018 |
| *O. ponerinarum* | HUA 186140 |  |  | KC610740 |  | KC610789 | KF658668 | Sanjuan et al. 2015 |
| *O. pruinosa* | NHJ 12994 |  | EU369041 | EU369024 | EU369084 | EU369106 | EU369063 | Johnson et al. 2009 |
| *O. pseudoacicularis* | TBRC 8101 |  | MF614645 | MF614629 | MF614676 |  | MF614662 | Luangsa-Ard et al. 2018 |
| *O. pseudocommunis* | BCC 16757 | MH754733 | MH753687 | MK284274 | MK214101 |  | MK214117 | Tasanathai et al. 2019 |
| *O. pseudolloydii* | MFLUCC 15**–**0689 | MF351725 |  | MF372758 |  |  |  | Xiao et al. 2017 |
| *O. pseudolloydii* | LHC | KX714602 |  | KX714603 |  |  |  | Chung et al. 2017 |
| *O. pseudorhizoidea* | BCC 48879 | MH754720 | MH753673 | MK284261 | MK214089 |  | MK214104 | Tasanathai et al. 2019 |
| *O. pulvinata* | TNS F 30044 |  |  | GU904209 |  | GU904208 | GU904210 | Quandt et al. 2014 |
| *O. purpureostromata* | TNS F18430 |  | KJ878897 | KJ878977 |  | KJ878931 | KJ879011 | Quandt et al. 2014 |
| *O. ramosissimum* | GZUHHN8 | KJ028007 |  | KJ028014 |  | KJ028012 | KJ028017 | Wen et al. 2014 |
| *O. ravenelii* | OSC 110995 |  | DQ518764 | DQ522334 | DQ522430 | DQ522550 | DQ522379 | Spatafora et al. 2007 |
| *O. rhizoidea* | NHJ 12522 | JN049857 | EF468825 | EF468764 | EF468923 | EF468970 | EF468873 | Sung et al. 2007a |
| *O. robertsii* | KEW 27083 |  | EF468826 | EF468766 |  |  |  | Sung et al. 2007a |
| *O. rubiginosiperitheciata* | NBRC 100946 | JN943341 | JN941436 | AB968581 | AB968543 | JN941705 | JN992439 | Ban et al. 2015 |
| *O. ryogamiensis* | NBRC 101751 |  | KF049633 | KF049688 |  | KF049614 | KF049650 | Kepler et al. 2013 |
| *O. satoi* | J7 |  | KX713599 | KX713683 |  | KX713653 | KX713711 | Araújo et al. 2018 |
| *O. satoi* | J19 |  | KX713601 | KX713684 |  | KX713650 | KX713710 | Araújo et al. 2018 |
| *O. sinensis* | ARSEF 6282 | KM652173 | KM652126 | KM652009 |  | KM652083 | KM652048 | Araújo et al. 2018 |
| *O. sinensis* | EFCC7287 | JN049854 | EF468827 | EF468767 | EF468924 | EF468971 | EF468874 | Sung et al. 2007a |
| *O. sobolifera* | KEW 78842 | JN049855 | EF468828 |  | EF468925 | EF468972 | EF468875 | Kepler et al. 2012 |
| *O. sobolifera* | TNS F18521 |  | KJ878898 | KJ878979 |  | KJ878933 | KJ879013 | Quandt et al. 2014 |
| *O. sp.* | Gh41 |  |  | KX713668 |  | KX713656 | KX713706 | Araújo et al. 2018 |
| *O. sp.* | TNS F18495 |  | KJ878901 |  |  | KJ878937 | KJ879017 | Quandt et al. 2014 |
| *O. sp.* | OSC 151904 |  | KJ878899 | KJ878980 |  | KJ878934 | KJ879014 | Quandt et al. 2014 |
| *O. sp.* | OSC 151905 |  |  | KJ878981 | KJ878951 | KJ878935 | KJ879015 | Quandt et al. 2014 |
| *O. sp.* | OSC 151909 |  | KJ878900 | KJ878982 | KJ878952 | KX713627 | KX713731 | Quandt et al. 2014 |
| *O. sp.* | FMF147 | KX197238 |  |  |  |  |  | Freire 2015 |
| *O. sp.* | OSC 110997 |  |  | EF468774 | EF468929 | EF468976 | EF468879 | Quandt et al. 2014 |
| *O. spataforae* | BCC 86480 |  | MG831747 | MG831746 | MG831749 |  | MG831748 | Luangsa-Ard et al. 2018 |
| *O. spataforae* | OSC 128575 | JN049845 | EF469079 | EF469064 | EF469110 | EF469126 | EF469093 | Sung et al. 2007a |
| *O. sphecocephala* | OSC 110998 |  | DQ518765 | DQ522336 | DQ522432 | DQ522551 | DQ522381 | Kepler et al. 2012 |
| *O. sphecocephala* | NBRC 101753 | JN943350 | JN941446 | AB968592 | AB968553 | JN941695 | JN992429 | Ban et al. 2015 |
| *O. sporangifera* | MFLUCC 18**–**0492 | MH725818 | MH725832 | MH727390 |  | MH725814 | MH727392 | Xiao et al. 2019 |
| *O. sporangifera* | MFLU 18**–**0658 | MH725817 | MH725831 | MH727389 |  | MH725813 | MH727391 | Xiao et al. 2019 |
| *O. stylophora* | OSC 110999 |  | EF468837 | EF468777 | EF468931 | EF468982 | EF468882 | Sung et al. 2007 |
| *O. stylophora* | OSC 111000 | JN049828 | DQ518766 | DQ522337 | DQ522433 | DQ522552 | DQ522382 | Spatafora et al. 2007 |
| *O. superficialis* | MICH 36253 |  |  |  |  | EF468983 | EF468883 | Sung et al. 2007a |
| *O. termiticola* | BCC 1920 | MH754724 | MH753678 | MK284265 | MK214094 |  | MK214108 | Tasanathai et al. 2019 |
| *O. termiticola* | BCC 1770 | GU723780 | MH753677 | MK284264 | MK214093 |  | MK214107 | Tasanathai et al. 2019 |
| *O. thanathonensis* | MFU 16**–**2910 | MF850375 | MF850378 | MF872614 |  | MF882926 | MF872616 | Xiao et al. 2017 |
| *O. thanathonensis* | MFU 16**–**2909 | MF850376 | MF850377 | MF872613 |  |  | MF872615 | Xiao et al. 2017 |
| *O. tiputini* | QCNE 186287 |  | KC610773 | KC610745 |  | KC610792 | KF658671 | Kepler et al. 2012 |
| *O. tricentri* | NBRC 106968 | AB968410 | AB968423 | AB968593 | AB968554 | AB968393 |  | Ban et al. 2015 |
| *O. unilateralis* | SERI1 |  | KX713626 | KX713675 |  | KX713628 | KX713730 | Araújo et al. 2018 |
| *O. unilateralis* | HUA 186161 |  |  | KC610742 | KC610718 | KC610799 | KF658674 | Sanjuan et al. 2015 |
| *O. unilateralis* | OSC 128574 |  | DQ518768 | DQ522339 | DQ522436 | DQ522554 | DQ522385 | Spatafora et al. 2007 |
| *O. variabilis* | ARSEF 5365 |  | DQ518769 | DQ522340 | DQ522437 | DQ522555 | DQ522386 | Kepler et al. 2012 |
| *O. variabilis* | OSC 111003 |  | EF468839 | EF468779 | EF468933 | EF468985 | EF468885 | Sung et al. 2007a |
| *O. vespulae* | GACP2017064 | MN044857 | MN044858 | MN117075 | MN107547 |  |  | Long et al.2020 |
| *O. vespulae* | GACP2017079 |  | MN044859 | MN117076 | MN107548 |  |  | Long et al.2020 |
| *O. xuefengensis* | GZUH2012HN11 | KC631800 |  | KC631791 |  | KC631786 | KC631796 | Wen et al. 2013 |
| ***O. aphrophoridarum*** | **MFLU 20–0641** | **MW139322** | **MW139330** | **MW160163** | **MW160167** | **MW139324** | **MW160165** | **This study** |
| ***O. aphrophoridarum*** | **MFLU 20–0642** | **MW139323** | **MW139331** | **MW160164** | **MW160168** | **MW139325** | **MW160166** | **This study** |
| *O. yakusimensis* | HMAS 199604 |  | KJ878902 |  | KJ878953 | KJ878938 | KJ879018 | Quandt et al. 2014 |
| *Tolypocladium inflatum* | OSC 71235 | JN049844 | EF469077 | EF469061 | EF469108 | EF469124 | EF469090 | Kepler et al. 2012 |
| *Tolypocladium ophioglossoides* | NBRC 106332 | JN943322 | JN941409 |  |  | JN941732 | JN992466 | Schoch et al. 2012 |
|  |  |  |  |  |  |  |  |  |
|  |  |  |  |  |  |  |  |  |
