# Supplementary material for: Ophiocordycepsaphrophoridarum sp. nov., a new entomopathogenic species from Guizhou, China
Source: Biodivers Data J. 2021 Dec 22;9:e66115. doi: 10.3897/BDJ.9.e66115 (PMC8716513; doi:10.3897/BDJ.9.e66115)
Supplement: Supplementary material 2 — References for GenBank accession numbers [file bdj-09-e66115-s002.docx]

References for GenBank accession numbers

Araújo JPM, Evans HC, Kepler R, Hughes DP (2018) Zombie–ant fungi across continents: 15 new speciesandnew combinations within *Ophiocordyceps*. I. Myrmecophilous hirsutelloid species. Studies in Mycology 90: 119–160. https://doi.org/10.1016/j.simyco.2017.12.002

Ban S, Sakane T, Nakagiri A (2015) Three new species of *Ophiocordyceps* and overview of anamorph types in the genus and the family Ophiocordyceptaceae. Mycological Progress 14(1): 1–12. https://doi.org/10.1007/s11557-014-1017-8

Castlebury LA, Rossman AY, Sung GH, Hyten AS, Spatafora JW (2004) Multigene phylogeny reveals new lineage for Stachybotrys chartarum, the indoor air fungus. Mycological Research 108(8): 864–872. https://doi.org/10.1017/S0953756204000607

Chen ZH, Dai YD, Yu H, Yang K, Yang ZL, Yuan F, Zeng WB (2013) Systematic analyses of *Ophiocordyceps lanpingensis* sp. nov., a new species of *Ophiocordyceps* in China. Microbiological Research 168(8): 525–532. http://dx.doi.org/10.1016/j.micres.2013.02.010

Chung TY, Sun PF, Kuo JI, Lee YI, Lin CC, Chou JY (2017) Zombie ant heads are oriented relative to solar cues. Fungal Ecology 25: 22–28. https://doi.org/10.1016/j.funeco.2016.10.003

Crous PW, Luangsa–Ard JJ, Wingfield MJ, Carnegie AJ, Hernandez–Restrepo M, Lombard L, Roux J, Barreto RW, Baseia IG, Cano–Lira JF, Martín MP (2018) Fungal Planet description sheets: 785–867. Persoonia: Molecular Phylogeny and Evolution of Fungi 41: 238. http://dx.doi.org/10.3767/persoonia.2018.41.12

Freire FM (2015) Taxonomia e distribuição de *Ophiocordyceps dipterigena* (Ophiocordycipitaceae, Hypocreales). Repositório Institucional da UFSC, 1–128. https://repositorio.ufsc.br/xmlui/handle/123456789/160715

Hyde K, Hongsanan S, Jeewon R, Bhat DJ, McKenzieE, Ebg J, Phookamsak R, Ariyawansa H, Boonmee S, Zhao Q, Abdel-Aziz F, Abdel-Wahab M, Banmai S, Chomnunti P, Cui B, Daranagama D, Das K, DayarathneM, Nl D, Zhu L (2016) Fungal diversity notes 367-490: taxonomic and phylogenetic contributions to fungal taxa.Fungal Diversity 80: 1-270. https://doi.org/10.1007/s13225-016-0373-x

Hyde K, Norphanphoun C, Pereira de Abreu V, Bazzicalupo A, Kandawatte T, Clericuzio M, Dayarathne M, Dissanayake A, Ekanayaka A, He MQ, Hongsanan S, Huang SK, Jayasiri S,Jayawardena R, Karunarathna A,Konta S,Kusan l, Lee H, Li Jf, Mortimer P (2017) Fungal diversity notes 603–708: taxonomic and phylogenetic notes on genera and species. Fungal Diversity 87(1): 1–235. https://doi.org/10.1007/s13225–017–0391–3

Hyde KD, Chaiwan N, Norphanphoun C, Boonmee S, Camporesi E, Chethana KWT, Dayarathne MC, de Silva NI, Dissanayake AJ, Ekanayaka AH, Hongsanan S (2018) Mycosphere notes 169–224. Mycosphere 9(2): 271–430. https://doi. 10.5943/mycosphere/9/2/8

Johnson D, Sung GH, Hywel-Jones NL, Luangsa-Ard JJ, Bischoff F, Kepler RM, Spatafora JW (2009) Systematics and evolution of the genus *Torrubiella* (Hypocreales, Ascomycota). Mycological Research 113 (3): 279‑289. https://doi.org/10.1016/j.mycres.2008.09.008

Kepler RM, Ban S, Nakagiri A, Bischoff J, Hywel–Jones N, Owensby CA, Spatafora JW (2013) The phylogenetic placement of hypocrealean insect pathogens in the genus *Polycephalomyces*: an application of one fungus one name. Fungal Biology 117(9): 611–622. https:// doi.org/10.1016/j.funbio.2013.06.002

Kepler RM, Sung GH, Ban S, Nakagiri A, Chen MJ, Huang B, Li Z, Spatafora JW (2012) New teleomorph combinations in the entomopathogenic genus *Metacordyceps*. Mycologia 104(1): 182–197. https://doi.org/10.3852/11–070

Li GJ, Hyde K, Zhao RL, Hongsanan S,Abdel-Aziz F, Abdel-Wahab M, Alvarado P, Silva G, AmmiratijJ,Ariyawansa H,Baghela A,Bahkali A, Beug M,Bhat D,Bojantchev D,Boonpratuang T, Bulgakov T, Erio Cc,Boro M, Amoozegar M(2016)Fungal diversity notes 253-366: taxonomic and phylogenetic contributions to fungal taxa. Fungal Diversity 78 https:/ldoi.org/10.1007/s13225-016-0366-9

Liu ZY, Liang ZQ, Liu AY, Yao YJ, Yu ZN (2002) Molecular evidence for teleomorph–anamorph connections in *Cordyceps* based on ITS–5.8S rDNA sequences. Mycological Research 106(9): 1100–1108. https://doi.org/10.1017/S0953756202006378

Long FY, Qin LW, Xiao YP, Hyde K, Wang SX, Wen TC (2021) Multigene phylogeny and morphology reveal a new species, *Ophiocordyceps vespulae*, from Jilin Province, China. Phytotaxa 478: 33‑48. https://doi.org/10.11646/phytotaxa.478.1.2

lto Y, Hirano T(1997)The determination of the partial 18 S ribosomal DNA sequences of *Cordyceps* species. Letters in Applied Microbiology 25: 239-42. https:/ldoi.org/10.1046/[j.1472-765X.1997.00203.x

Luangsa–Ard JJ, Ridkaew R, Tasanathai K, Thanakitpipattana D, Hywel–Jones N (2011) *Ophiocordyceps halabalaensis*: a new species of *Ophiocordyceps* pathogenic to *Camponotus gigas* in Hala Bala Wildlife Sanctuary, Southern Thailand. Fungal Biology 115(7): 608–614. https://doi.org/10.1016/j.funbio.2011.03.002

Luangsa–Ard JJ, Tasanathai K, Thanakitpipattana D, Khonsanit A, Stadler M (2018) Novel and interesting *Ophiocordyceps* spp. (Ophiocordycipitaceae, Hypocreales) with superficial perithecia from Thailand. Studies in Mycology 89: 125–142. https://doi.org/10.1016/j.simyco.2018.02.001

Luangsa–Ard JJ, Ridkaew R, Mongkolsamrit S, Tasanathaib K, Hywel–Jonesb NL (2010) *Ophiocordyceps barnesii* and its relationship to other melolonthid pathogens with dark stromata. Fungal Biology 114(9): 739–745. https://doi.org/10.1016/j.funbio.2010.06.007

Quandt C, Kepler R, Araujo J,Ban S, Evans H, Hughes D, Hywel-Jones N, Li ZZ, Luangsa-Ard J, Rehner S, Sanjuan T, Sato H, Shrestha B, Sung GH, Yao YJ,Zare R, Spatafora J (2014) Phylogenetic-based nomenclatural proposals for Ophiocordycipitaceae (Hypocreales) with new combinations in *Tolypocladium*. IMA Fungus 5 https://doi.org/10.5598/imafungus.2014.05.01.12

Saltamachia S, Araujo JM (2020) *Ophiocordyceps desmidiospora*, a basal lineage within the "Zombie-Ant Fungi" clade. Mycologia 112(6): 1171-1183. https:/ldoi.org/10.1080/00275514.2020.1732147

Sanjuan TI, Franco–Molano AE, Kepler RM, Spatafora JW, Tabima J, Vasco–Palacios AM, Restrepo S (2015) Five new species of entomopathogenic fungi from the Amazon and evolution of neotropical *Ophiocordyceps.* Fungal Biology 119(10): 901–916. https://doi.org/10.1016/j.funbio.2015.06.010

Schoch CL, Seifert KA, Huhndorf S, Robert V, Spouge JL, Levesque CA, Chen W, Bergeron MJ, Hamelin RC, Vialle A, Fungal Barcoding Consortium. (2012) Nuclear ribosomal internal transcribed spacer (ITS) region as a universal DNA barcode marker for Fungi. Proceedings of the National Academy of Science 109: 6241–6246. https://doi: 10.1073/pnas.1117018109

Simmons DR, Lund J, Levitsky T, Groden E(2015) *Ophiocordyceps myrmicarum*, a new species infecting invasive *Myrmica rubra* in Maine. Journal of lnvertebrate Pathology 125:23-30. https:/ldoi.org/10.1016/jjip.2014.12.010

Spatafora JW, Sung GH, Sung JM, Hywel–Jones NL, White JJF (2007) Phylogenetic evidence for an animal pathogen origin of ergot and the grass endophytes. Molecular Ecology 16(8): 1701–1711. https://doi.org/10.1111/j.1365–294X.2007.03225.x

Suh SO, Spatafora J, Ochiel G, Evans H,Blackwell M(1998). Molecular phylogenetic study of a termite pathogen *Cordycepioideus bisporus*. Mycologia 90: 611‑617. https://doi.org/10.1080/00275514.1998.12026950

Sung GH, Hywel–Jones NL, Sung JM, Luangsa–ard JJ, Shrestha B, Schoch JW (2007a) A multi-gene phylogeny of Clavicipitaceae (Ascomycota, Fungi): Identification of localized incongruence using a combinational bootstrap approach. Molecular Phylogenetics and Evolution 44: 1204‑23. https://doi.org/10.1016/j.ympev.2007.03.011

Tasanathai K, Noisripoom W, Chaitika T, Khonsanit A, Hasin S, Luangsa-Ard J (2019) Phylogenetic and morphological classification of *Ophiocordyceps* species on termites from Thailand. MycoKeys 56: 101‑129. <https://doi.org/10.3897/mycokeys.56.37636>

Torres FZV, Souza D, Da Costa Lira E, Faria M, Sujii E and Lopes RB (2018) Occurrence of the anamorphic stage of *Ophiocordyceps myrmicarum* on a non-Formicidae insect in integrated crop-livestock farming systems. Fungal Ecology 34: 83‑90. https://doi.org/10.1016/j.funeco.2018.05.009

Wen TC, Xiao YP, Li WJ, Kang JC, Hyde KD (2014) Systematic analyses of *Ophiocordyceps ramosissimum* sp. nov., a new species from a larvae of Hepialidae in China. Phytotaxa 161(3): 227–234. http://dx.doi.org/10.11646/phytotaxa.161.3.6

Wen TC, Zhu RC, Kang JC, Huang MH, Tan DB, Ariyawansha H, Hyde KD, Liu H (2013) *Ophiocordyceps xuefengensis* sp. nov. from larvae of *Phassus nodus* (Hepialidae) in Hunan Province, southern China. Phytotaxa 123(1): 41–50. http://dx.doi.org/10.11646/phytotaxa.123.1.2

Xiao YP, Hongsanan S, Hyde KD, Brooks S, Xie N, Long FY, Wen TC (2019) Two new entomopathogenic species of *Ophiocordyceps* in Thailand. MycoKeys 47: 53–74. http://dx.doi.org/10.3897/mycokeys.47.29898

Xiao YP, Wen TC, Hongsanan S, Sun JZ, Hyde KD (2017) Introducing *Ophiocordyceps thanathonensis*, a new species of entomogenous fungi on ants, and a reference specimen for *O. pseudolloydii*. Phytotaxa 328(2): 115–126. http://dx.doi.org/10.11646/phytotaxa.328.2.2

Yang ZL, Qin J, Xia C, Hu Q, Li QQ, Yang ZL (2015) *Ophiocordyceps highlandensis*, a new entomopathogenic fungus from Yunnan, China. Phytotaxa 204(4): 287–295. http://dx.doi.org/10.11646/phytotaxa.204.4.5

Zhang WM, Wang L,Tao MH,Chen YQ,Qu LH (2007) Two species of *Cordyceps* simultaneously parasitic ona larva of Lepidoptera. Mycosystema 26.
